# Enterovirus 68 in Children with Acute Respiratory Tract Infections, Osaka, Japan

**DOI:** 10.3201/eid1708.110028

**Published:** 2011-08

**Authors:** Atsushi Kaida, Hideyuki Kubo, Jun-ichiro Sekiguchi, Urara Kohdera, Masao Togawa, Masashi Shiomi, Toshinori Nishigaki, Nobuhiro Iritani

**Affiliations:** Author affiliations: Osaka City Institute of Public Health and Environmental Sciences, Osaka, Japan (A. Kaida, H. Kubo, J. Sekiguchi, N. Iritani);; Nakano Children’s Hospital, Osaka (U. Kohdera);; Osaka City Sumiyoshi Hospital, Osaka (M. Togawa);; Osaka City General Hospital, Osaka (M. Togawa, M. Shiomi);; Osaka Police Hospital, Osaka (T. Nishigaki)

**Keywords:** Enterovirus 68, enterovirus, viruses, acute respiratory tract infections, febrile convulsions, respiratory infections, Japan, children, infants, dispatch

## Abstract

Enterovirus 68 strains were detected in 14 specimens from children with respiratory tract infections and 1 specimen from a child with febrile convulsions during 2010 in Osaka, Japan. These strains had deletions in the 5′ untranslated region and were genetically different from reported strains. This virus is associated with respiratory tract infections in Japan.

Enterovirus 68 (EV68) belongs to the family *Picornaviridae*, genus *Enterovirus*, and species *Human enterovirus D* (*1*). EV68 was isolated from 4 children with pneumonia and bronchiolitis in the United States in 1962 (prototype Fermon strain) (*2**,**3*) and is associated with respiratory tract infections (RTIs) (*3**–**5*). The most common age group for infection with EV68 is 1–4 years of age, but ≈25% of EV68 cases occur in adults >20 years of age (*5*).

Because of its acid sensitivity and low optimum growth temperature (33°C), EV68 shares characteristics with human rhinovirus (HRV) (*3**,**6*) and is genetically and antigenically similar to HRV 87 (*6**,**7*). During 1970–2005, only 26 EV68 strains were detected in the United States (*5*). Fourteen detections of EV68 were reported during 2006–2009 in Japan: 2 in 2006, 8 in 2007, and 4 in 2009 (*8*). EV68 is rarely detected in Japan, and no epidemics have been reported. We report deletions in genomes of EV68 strains detected in Japan.

## The Study

During October 2009–October 2010, a total of 448 respiratory specimens were obtained from 448 patients (258 male patients and 190 female patients) with RTIs and fevers in a virus surveillance system in Osaka, Japan (*9*). The mean ± SD age of the patients was 41.4 ± 53.7 months (range <1–404 months), and 351 (78.3%) were <5 years of age.

Procedures for viral nucleic acid extraction and cDNA synthesis have been reported (*9*). PCR for detecting HRV and enterovirus was conducted by using EVP4 and OL68-1 primers, which detected HRV and human enterovirus, respectively, in amplicons of ≈530 and 650 bp, respectively (*7*).

Results showed 178 positive specimens (140 for HRV, 16 for human enterovirus, 7 for HRV and human enterovirus, and 15 for an unexpected amplicon of ≈600 bp). To identify the 600-bp amplicon, we sequenced viral protein 4 (VP4) and VP1 genes. BLAST analysis (www.ncbi.nlm.nih.gov/) showed that these isolates had high identity with EV68 VP4 (98.5%–99.5% with the Pav254–26868 strain [GenBank accession no. HM370293]) and VP1 (96.7%–97.5% with the MD02–1 strain [GenBank accession no. AY426491]). Therefore, these isolates were EV68 positive.

**Figure 1:**
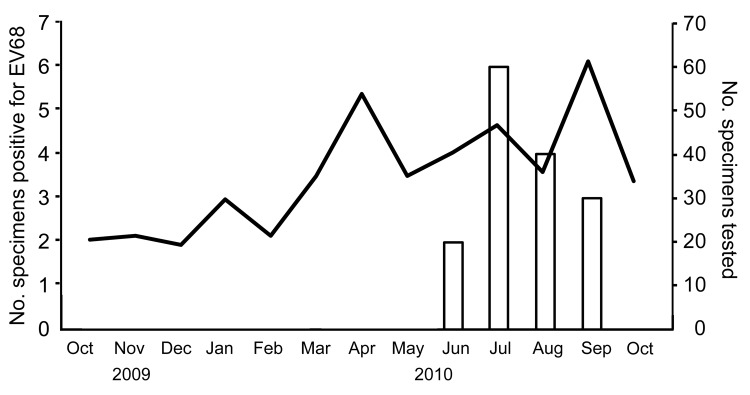
Monthly distribution of enterovirus 68 (EV68) in Osaka, Japan, October 2009–October 2010, Bars indicate no. specimens positive for EV68 and line indicates no. specimens tested.

**Figure 2:**
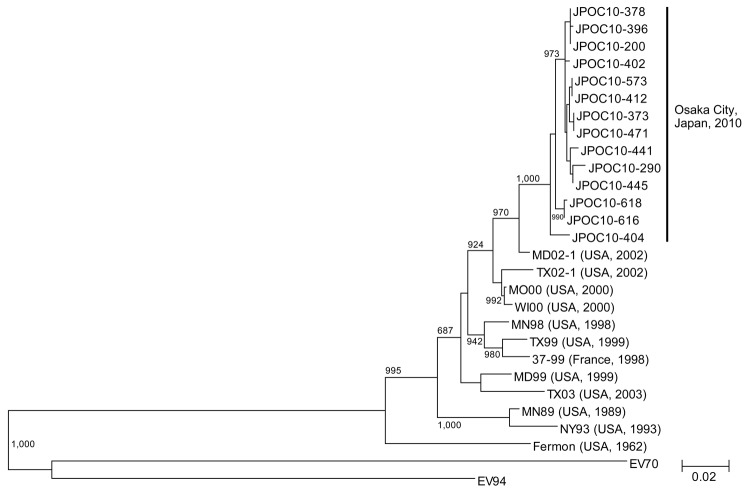
Phylogenetic tree of enterovirus 68 viral protein 1 gene sequences constructed by using a 927-nt sequence corresponding to nt sequence 2355–3281 in strain 37–99, Osaka, Japan, October 2009–October 2010. Tree was constructed by using the neighbor-joining method. Sequences were aligned by using Clustal X version 1.81. (www.clustal.org/). Genetic distances between sequences were calculated by using the Kimura 2-parameter method. Bootstrap values from 1,000 replicates are shown at the nodes. Location and year of collection are shown in parentheses. GenBank accession numbers for strains used in this analysis were Fermon, AY426531; 37–99, EF107098; JPOC10–200, AB601872; JPOC10–290, AB601882; JPOC10–373, AB601873; JPOC10–378, AB601883; JPOC10–396, AB601884; JPOC10–402, AB601874; JPOC10–404, AB601885; JPOC10–412, AB601875; JPOC10–441, AB601876; JPOC10–445, AB601877; JPOC10–471, AB601878; JPOC10–573, AB601879; JPOC10–616, AB601880; JPOC10–618, AB601881; MD02–1, AY426491; TX02–1, AY426495; MO00, AY426493; WI00, AY426494; MN98, AY426497; TX99, AY426498; MD99, AY426499; TX03, AY426500; MN89, AY426489; NY93, AY426490; EV70, D00820; and EV94, DQ916376. Scale bar indicates nucleotide substitutions per site.

EV68 was not detected in virus isolation tests with Vero and RD-18S cells. EV68 was detected during June–September 2010 (Figure 1). Characteristics of 15 EV68-positive patients are shown in Table 1. Phylogenetic analysis using VP1 sequences (14 of 15 Osaka strains were sequenced) demonstrated that Osaka strains were clustered in 1 group and differed from previously reported strains (Figure 2).

**Table 1:** Characteristics of 15 patients in whom enterovirus 68 was detected, Osaka, Japan, 2010

Patient no.	Age/sex	Sample no.	Month sampled	Clinical samples	Diagnosis or signs
1	4 y 9 mo/M	JPOC10–200	Jun	Nasal mucus	Bronchitis, fever (38.7°C), wheezing, coughing
2	0 y 7 mo/F	JPOC10–290	Jun	Nasal mucus	Asthmatic bronchitis, fever (39.3°C)
3	3 y 6 mo/M	JPOC10–373	Jul	Nasal mucus	Asthmatic bronchitis, fever (37.4°C)
4	2 y 10 mo/F	JPOC10–378	Jul	Nasal mucus	Pneumonia, fever (40°C), pharyngitis
5	4 y 6 mo/F	JPOC10–396	Jul	Nasal mucus	Pneumonia, wheezing, dyspnea
6	5 y 0 mo/F	JPOC10–402	Jul	Nasal mucus	Asthma, respiratory failure
7	1 y 8 mo/M	JPOC10–404	Jul	Nasal mucus	Febrile convulsion, fever (39°C)
8	3 y 9 mo/M	JPOC10–412	Jul	Sputum	Lower respiratory tract infection, fever (39°C)
9	1 y 3 mo/F	JPOC10–441	Aug	Nasal mucus	Asthmatic bronchitis, fever (38.2°C), wheezing
10	4 y 1 mo/M	JPOC10–445	Aug	Sputum	Asthmatic bronchitis
11	1 y 6 mo/M	JPOC10–471	Aug	Nasal mucus	Bronchopneumonia, fever (39°C)
12	0 y 3 mo/M	JPOC10–515	Aug	Throat swab	Pharyngitis, fever (38°C)
13	3 y 5 mo/M	JPOC10–573	Sep	Throat swab	Asthmatic bronchitis, fever
14	0 y 7 mo/M	JPOC10–616	Sep	Nasal mucus	Asthmatic bronchitis
15	1 y 5 mo/M	JPOC10–618	Sep	Nasal mucus	Pneumonia, fever (39°C)

Nucleotide and amino acid identities among 4 Osaka EV68 (JPOC10-290, 378, 396, and 404 strains; nt 501–7265 corresponding to the 37–99 strain), Fermon, and 37–99 strains were determined. To determine sequences, we synthesized cDNA by using specific primers and amplified 4 segments (nt 160–1153, 543–3391, 3132–4032, and 3747–7333 corresponding to the 37–99 strain). The Fermon and 37–99 strains are the only EV68 strains for which complete genome sequences are available. The 37–99 strain was isolated from a 6-year-old girl with pneumonia in 1998 (H. Norder, pers. comm.). The sequenced region coded partial 5′ untranslated regions (UTRs) and all structural and nonstructural viral proteins (VP4–3D). Identities between strains were calculated by using BioEdit version 7.09 (www.mbio.ncsu.edu/bioedit/bioedit.html) (Table 2).

**Table 2:** Comparison of nucleotide and amino acid sequences of Osaka enterovirus 68 strains detected in Osaka, Japan, October 2009–October 2010, with Fermon and 37–99 strains*

Region	Nucleotide identity, %				Amino acid identity, %		
	Osaka strains	Fermon	37–99		Osaka strains	Fermon	37–99
VP4–3D	98.4–99.6	87.8–87.9	93.9–94.1		99.4–99.8	95.8–96.0	98.4–98.7
VP4	97.5–100	90.8–91.7	94.6–95.6		100	100	100
VP2	97.5–99.7	87.3–87.7	94.4–94.6		100	94.3	99.1
VP3	97.7–99.7	85.5–85.8	93.9–94.4		99.5–100	94.0–94.4	98.7–99.1
VP1	97.7–99.8	87.5–87.8	92.5–93.2		99.0–100	90.6–90.9	95.4–96.1
2A	96.5–99.7	86.6–87.5	93.1–93.6		98.6–100	97.2–97.9	99.3–100
2B	96.6–99.6	83.5–85.8	91.9–92.5		98.9–100	96.9–97.9	97.9–98.9
2C	98.7–99.6	89.0–89.2	94.5–94.7		100	97.8	99
3A	99.6–100	89.5–89.8	94.0–94.3		98.8–100	96.6–97.7	98.8–100
3B	98.4–100	87.8–89.3	96.9–98.4		100	100	100
3C	98.2–99.3	87.0–87.7	92.4–93.2		99.0–100	96.2–96.7	98.1–98.5
3D	99.0–99.6	88.6–88.9	94.6–95.0		99.0–100	97.4–97.8	98.3–99.0

*Osaka strains, JPOC10-290, JPOC10-378, JPOC10-396, and JPOC10-404; VP, viral protein.

Among Osaka strains, nucleotide and amino acid sequences were highly conserved (nt identity 96.5%–100% and aa identity 98.6%–100%). In contrast, Osaka strains had lower similarities with the Fermon strain (nt identity 83.5%–91.7% and aa identity 90.6%–100%) than with the 37–99 strain (nt identity 91.9%–98.4% and aa identity 95.4%–100%). When we compared individual viral proteins in Osaka strains with those in the Fermon strain, no gene except for VP4 showed >90% nt sequence identity; gene 2B showed the lowest identity (83.5%–85.8%). In contrast, the 37–99 strain had >91.9% nt identity with Osaka strains.

Regarding amino acids, <95% identity was observed in VP1, VP2, and VP3 in the Fermon strain, and no genes with <95% aa identity were found in the 37–99 strain in contrast with Osaka strains. Moreover, no integration or deletion of nucleotides was observed in VP4–3D sequences among Osaka, Fermon, and 37–99 strains.

**Figure A1:**
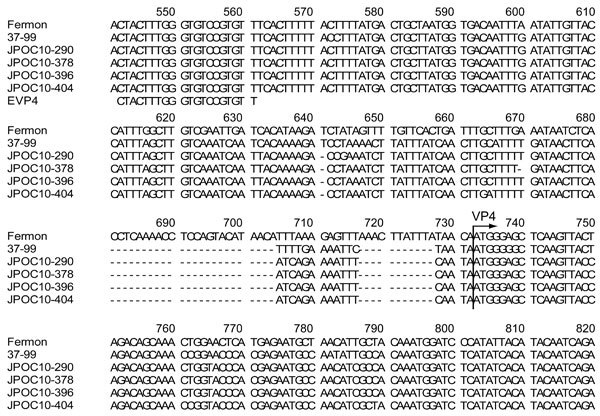
Nucleotide sequences of 5′ untranslated regions of enterovirus 68 strains, Osaka, Japan, October 2009–October 2010. The strain detected in Osaka showed deletions when compared with Fermon and 37–99 strains. Arrow indicates the start codon (ATG) of the viral protein 4 (VP4) gene. Partial nucleotide sequences (corresponding to nt 541–820 of the Fermon strain) of 5′ untranslated regions of Fermon, 37–99, JPOC10–290, JPOC10–378, JPOC10–396, JPOC10–404, and EVP4 primer were aligned by using BioEdit version 7.09 (www.mbio.ncsu.edu/bioedit/bioedit.html). Hyphens indicate deleted nucleotides.

To clarify why EV68 Osaka strain genomes were smaller than those of other enteroviruses and the EV68 Fermon strain (*7*), we aligned the partial 5′ UTR sequences (nt 541–820 corresponding to the Fermon strain) of 4 Osaka, Fermon, and 37–99 strains (Figure A1). Results showed that the Osaka and 37–99 strains had deletions at nt 681–704 and 717–727 in contrast with the Fermon strain. Moreover, a 1-nt deletion in Osaka strains was identified at nt 641 in contrast with the Fermon and 37–99 strains. Only the JPOC10–378 strain had a 1-nt deletion at nt 670.

## Conclusions

Because 14 patients with EV68 were detected during 2006–2009 (*8*), detection of 15 patients with EV68 during a 4-month period suggests an EV68 epidemic in the summer of 2010 in Japan. Phylogenetic analysis with VP1 sequences showed that Osaka strains differed genetically from previously reported strains.

For precise analysis of Osaka, Fermon, and 37–99 strains, nucleotide and amino acid sequences were compared in all viral proteins. Results showed that Osaka strains more closely resembled the 37–99 strain than the Fermon strain. Alignment of partial 5′ UTR sequences showed that Osaka and 37–99 strains had deletions in 2 regions in contrast with the Fermon strain, and the amplicon was shorter than expected. Moreover, Osaka strains had 1-nt deletions in contrast with the 37–99 strain.

The 5′ UTR of enterovirus contains an internal ribosome entry site (*10*) that is associated with translational efficiency and virulence of the enterovirus (*11**,**12*). Deleted regions of Osaka strains appear to be in the flanking region between the internal ribosome entry site and an open reading frame (*1*). Detection of EV68 in numerous patients was reported in France during 2008 (*4*) and Italy during 2008–2009 (*13*). Because this deletion was found in the 37–99 strain in 1998, recent detection of EV68 in Japan might be associated with this change in the viral genome. Smura et al. reported that serum samples from 281 pregnant women in Finland in 1983, 1993, and 2002 had high titers of neutralizing antibody against EV68 (*14*). This result indicates that EV68 has been in Finland since 1983.

All EV68-positive patients in this study were <5 years of age and had lower respiratory tract inflammation. Seroepidemiologic studies in Finland showed that most adults might have been previously infected with EV68 and therefore might have neutralizing antibodies (*14*). Increased detection of EV68, especially in infants and children, will provide useful epidemiologic data.

Recent studies showed that EV68-infected human leukocytes produced infectious progeny virus (*14*). This result indicates that EV68 can replicate in blood and may damage the central nervous system. EV68 was detected in cerebrospinal fluid of a young adult patient with acute flaccid paralysis (*5*). Epidemiologic data for EV68 are lacking, and little information is available regarding virologic characteristics. If one considers results of phylogenetic analyses and nucleotide and amino acid identities, evolutionary changes might have occurred in EV68. Our results show the potential role of EV68 infection in infants and children with RTIs.
